# Pain relief and improvement in quality of life with 10 kHz SCS therapy: Summary of clinical evidence

**DOI:** 10.1111/cns.13285

**Published:** 2020-02-22

**Authors:** Dawood Sayed, Jan Willem Kallewaard, Anand Rotte, Jessica Jameson, David Caraway

**Affiliations:** ^1^ University of Kansas School of Medicine Kansas City KS USA; ^2^ Department of Anesthesiology and Pain Medicine Rijnstate Hospital Arnhem Velp The Netherlands; ^3^ Nevro Corp. Redwood City CA USA; ^4^ Axis Spine Center Post Falls ID USA

**Keywords:** 10 kHz SCS, chronic pain, opioids, quality of life, VAS

## Abstract

**Objective:**

Chronic pain is a prevalent condition which has a significant effect on the lives of those it impacts. High‐frequency 10 kHz spinal cord stimulation (10 kHz SCS) has been shown to provide paresthesia‐free pain relief for a wide variety of pain indications. This article summarizes the current and emerging data as they relate to the clinical use of the therapy in various pain syndromes.

**Methods:**

A literature search was conducted using the PubMed electronic database using keywords related to 10 kHz SCS. The database was queried from 2013 to May 2019. Articles reporting clinical studies that included human subjects permanently treated with 10 kHz SCS (Senza® system) were included in the review. Recent and relevant conference proceedings known to the authors were also included.

**Results:**

The selected literature demonstrated significant evidence for the efficacy of 10 kHz SCS in treating chronic back and leg pain (CBLP), including a randomized, controlled trial as well as prospective and retrospective studies. One‐year follow‐up responder rates (pain relief ≥50%) ranged from 60% to 80%. Other studies and case series showed promising outcomes in specific conditions, including nonsurgical refractory back pain, neuropathic limb pain, complex regional pain syndrome, chronic widespread pain, chronic pelvic pain, and intractable headache. Subgroup analyses also pointed toward the potential of 10 kHz SCS being successful when low‐frequency SCS has failed. The vast majority of these studies reported improved quality of life (QOL) metrics and/or reduced opioid consumption.

**Conclusions:**

Level I evidence already exists for the efficacy of 10 kHz SCS in treating CBLP, supported by real‐world clinical experience. Other studies demonstrate the potential of the therapy across a range of chronic pain etiologies, although larger confirmatory studies are recommended. Overall, the literature suggests that the therapy is associated with improved QOL as well as reduced opioid consumption.

## 1 INTRODUCTION

Chronic pain is an escalating public health issue around the world. It represents a significant challenge to individuals and their families, as well as healthcare providers and payers. Surveys estimate that around one in five adults report chronic pain.^1^, ^2^, ^3^ Some studies indicate that the prevalence increases with age and is rising overall.^1^, ^4^, ^5^, ^6^, ^7^, ^8^, ^9^, ^10^ Chronic pain is known to have a major impact on patients’ well‐being, relationships with others, ability to carry out everyday activities, and work productivity.^3^, ^11^, ^12^, ^13^, ^14^, ^15^, ^16^, ^17^, ^18^, ^19^ The associated economic burden to society exceeds $500 billion annually in the United States and consumes 2%‐10% of GDP in European countries.^20^, ^21^, ^22^, ^23^

The treatment of chronic pain is complex and challenging, encompassing many disciplines, including physical and psychological therapies, as well as pharmacological, interventional, and surgical treatments.^24^ Opioid medication is often prescribed as part of a pain management strategy. While there is some evidence supporting the short‐term use of opioids in chronic pain, long‐term efficacy data are lacking.^25^, ^26^, ^27^ For a variety of reasons, long‐term opioid prescribing has been stable nonetheless.^28^ Traditional low‐frequency spinal cord stimulation (LF‐SCS) is another therapeutic option for chronic pain and is most commonly used to treat leg pain associated with failed back surgery syndrome (FBSS).^29^, ^30^ The therapy has Level I‐II efficacy evidence in this indication as well as established cost‐effectiveness.^31^, ^32^, ^33^, ^34^, ^35^, ^36^, ^37^ Despite the robust body of evidence in favor of its use in FBSS, the therapy is arguably underutilized with the vast majority of patients undergoing spinal reoperation (>97%) despite poor published success rates.^37^, ^38^

During LF‐SCS, one or more thin percutaneous leads or a surgical paddle lead with integrated electrical contacts are placed in the epidural space of the spinal canal. The vertebral level is selected according to the area of pain and is mapped via patient feedback for each patient. Electrical pulses to the spinal cord are applied via a temporary or permanently implanted pulse generator at a fixed frequency, usually in the range of 40‐60 Hz with a pulse width 150‐500 µs.^39^ Historically, it is understood that approximately half of patients achieve ≥50% pain relief.^31^, ^32^ However, some report uncomfortable paresthesia or experience discomfort related to under‐ or overstimulation resulting from postural changes.^40^, ^41^, ^42^ Additionally, paresthesia can be difficult to isolate in very common regions of chronic pain such as the low back and foot. In some patients, habituation occurs, and pain relief can diminish after several years.^43^, ^44^, ^45^, ^46^, ^47^, ^48^

While several technological advances in LF‐SCS have been made during the last decade, these gains have not translated into higher success rates.^33^ Consequently, efforts have been made to develop new systems with more advanced stimulation waveforms. One such system, 10 kHz SCS (Senza® system), has been developed by Nevro Corp. Along with a higher stimulation frequency compared with LF‐SCS, it utilizes a shorter pulse width (30 µs) and lower amplitude electrical pulses (1.0‐5.0 mA).^33^ Patients do not experience paresthesia. Leads are implanted along the anatomical midline with lead tips located at T8 and T9 in a staggered fashion covering T8‐T11 vertebral levels for back and leg pain patients. The implantation procedure is more predictable and reproducible than that required for LF‐SCS due to the absence of paresthesia mapping and associated patient feedback.^49^ In comparison with LF‐SCS, where paresthesia mapping during the procedure is mandatory, leads are placed anatomically during 10 kHz SCS implantations without paresthesia mapping.

Over the last 5 years, a considerable evidence base has been established for the clinical use of 10 kHz SCS for the treatment of chronic pain in the trunk and/or limbs. Evidence has also been emerging for its utility in treating intractable headache as well as other common pain syndromes with limited therapeutic options. This purpose of this article is to provide a comprehensive summary of prospective and retrospective clinical studies in the field, as well as ongoing investigations, with a specific emphasis on clinical outcomes, including pain relief as well as changes in quality of life (QOL) and opioid consumption (Table 1).

**Table 1:** List of prospective and retrospective studies evaluating benefits of 10 kHz SCS

References	Study type	Key inclusion	N*	FU period	Outcomes
Kapural et al (2015)^33^; Kapural et al (2016)^51^; Amirdelfan et al (2018)^50^	Multicenter RCT	≥5 pts VAS back and leg	90	24 mo	VAS, responder rate, remitter rate, trial‐to‐perm ratio, changes in medication use, ODI, GAF, SF‐MPQ‐2, SF‐12, CGIC, PSQI, and satisfaction
Stauss et al (2019)^55^	Retrospective, multicenter, review	Back and leg pain	1660	12 mo	VNRS, responder rate, trial‐to‐perm ratio, changes in medication use, general function, general QOL & sleep, and satisfaction
VanBuyten et al (2013)^52^; Al‐Kaisy et al (2014)^53^	Prospective, two‐center	Primary diagnosis of chronic back pain	72	24 mo	VAS, responder rate, trial‐to‐perm ratio, changes in medication use, ODI, sleep disturbance, and satisfaction
Al‐Kaisy et al (2017)^57^; Al‐Kaisy et al (2018)^58^	Prospective, single‐center	Predominant chronic back pain, no history of/eligibility for spinal surgery	20	36 mo	VAS, responder rate, trial‐to‐perm ratio, changes in medication use, ODI, SF‐36 PC & MC EQ5D TTO, QALY gain, sleep disturbance, and satisfaction
Al‐Kaisy et al (2015)^59^	Single‐center, retrospective, case series	Neuropathic pain upper or lower limbs	11	6 mo	NRS, responder rate, trial‐to‐perm ratio, BPI, PCS, EQ‐5D, painDETECT, and satisfaction
Gill et al (2019)^60^	Single‐center, retrospective, review	Uni‐ or bilateral CRPS	12	12.1 ± 4.6 mo	NRS, responder rate, trial‐to‐perm ratio, and SF‐MPQ‐2
Salmon (2019)^64^	Single‐center, retrospective, review	Combined upper and lower body neuropathic/nociplastic pain syndromes	38	2.3 ± 1.7 y	NRS, trial‐to‐perm ratio, changes in medication use, RMDQ, PGIC, PSEQ, DASS, and satisfaction
Russo et al (2016)^61^	Multicenter, retrospective, review	Not candidates for SCS or nonresponders	189	6 mo	NPRS, responder rate, trial‐to‐perm ratio, and ODI
Arcioni et al (2016)^66^	Single‐center, prospective, open‐label	rCM**	15	6 mo	Headache days, trial‐to‐perm ratio, changes in medication use, MIDAS, and HIT‐6
Lambru et al (2016)^68^	Single‐center, retrospective, case series	rCM**	4	25.3 mo	Headache days, trial‐to‐perm ratio, changes in medication use, HIT‐6
Amirdelfan et al NANS 2019 Annual Meeting^69^	Multicenter, prospective, open‐label	Upper limb and/or neck pain**	45	12 mo	VAS, responder rate, remitter rate, trial‐to‐perm ratio, PDI, and SF‐MPQ‐2
Galan et al NANS 2019 Annual Meeting^72^	Multicenter, prospective, open‐label	Peripheral polyneuropathy upper or lower limbs	18	24 mo	VAS, responder rate, trial‐to‐perm ratio, PDI, and SF‐MPQ‐2
Tate et al NANS 2019 Annual Meeting^75^	Multicenter, prospective, open‐label	Chronic pelvic pain	17	12 mo	VAS, responder rate, remitter rate, trial‐to‐perm ratio, PDI, and SF‐MPQ‐2
Gupta et al NANS 2019 Annual Meeting^73^	Multicenter, prospective, open‐label	Postsurgical pain trunk and/or limbs	28	12 mo	VAS, responder rate, trial‐to‐perm ratio, PDI, and SF‐MPQ‐2
Burgher et al NANS 2019 Annual Meeting^71^	Multicenter, prospective, open‐label	Upper extremity pain	33	12 mo	VAS, responder rate, trial‐to‐perm ratio, PDI, QuickDASH, GAF, PSQ3, and satisfaction
Kapural et al NANS 2019 Annual Meeting^74^	Multicenter, prospective, open‐label	Abdominal pain**	22	12 mo	VAS, responder rate, trial‐to‐perm ratio, PSQ3, and PGIC

Note

FU‐follow‐up; N*‐number of implanted subjects/patients; **off‐label indications for SCS.

## 2 METHODS

A literature search was conducted using the PubMed electronic database using keywords related to 10 kHz SCS, such as spinal cord stimulation, 10 kHz, and HF10. The database was queried from 2013 to May 2019. Results were limited to English‐language articles reporting clinical studies that included human subjects permanently treated with 10 kHz SCS (Senza® system). Recent and relevant conference proceedings known to the authors were also included in the review.

## 3 RESULTS

### 3.1 Chronic back and leg pain

#### 3.1.1 SENZA‐RCT study

**Figure 1:**
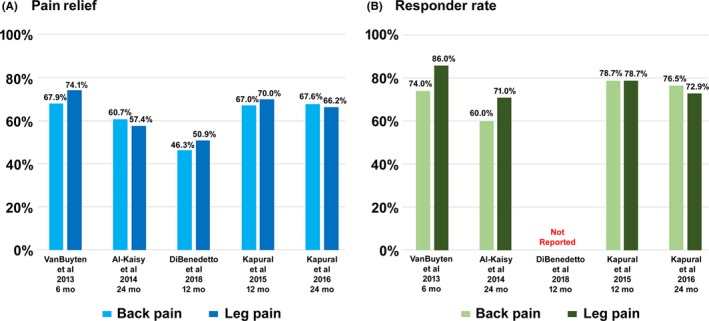
10 kHz SCS benefits for low back and leg pain patients. A, Mean pain relief B, Responder rate

A pivotal, multicenter, randomized, controlled trial (RCT) published in 2015 by Kapural et al established Level I evidence for the efficacy of 10 kHz SCS in treating chronic back and leg pain (SENZA‐RCT).^33^ The study inclusion criteria specified both back and leg pain scores ≥5 cm on the visual analog scale (VAS). Subjects were randomly assigned (1:1) to receive either 10 kHz SCS or traditional LF‐SCS. Most had undergone previous spinal surgery (87%) while just over half of each group reported predominant back pain. Ninety of 97 subjects (93%) in the 10 kHz SCS group and 81 of 92 subjects (88%) in the LF‐SCS group completed a successful trial and received a permanent system. Outcomes were compared up to 12 months postimplantation. Response to therapy was defined as ≥50% reduction in pain score. At the 3‐month primary endpoint, 84% and 83% of the 10 kHz SCS group were responders for back pain and leg pain, respectively, compared with 44% and 55% of the LF‐SCS group (*P* < .001 for both noninferiority and superiority in both pain categories). At 12 months, outcomes were available for 89 and 80 subjects in the 10 kHz SCS and LF‐SCS groups, respectively. Responder rates were sustained in both groups and pain categories but remained higher in the 10 kHz SCS group (back pain: 79% vs 51%; leg pain: 79% vs 51%, *P* < .001 for both noninferiority and superiority in both pain categories). Moreover, at the same time point, the 10 kHz SCS group reported a decrease in back pain and leg pain of 67% and 70%, respectively (Figure 1), compared with 44% and 49% in the LF‐SCS group (back pain: −4.9 vs −3.5 cm; leg pain: −5.0 vs −3.8 cm, *P* < .001 in both pain categories). Several secondary outcome measures also favored 10 kHz SCS subjects at 12 months. The 10 kHz SCS group reported a larger decrease in morphine equivalent daily dose (MEDD: −24.8 vs −7.3 mg/d, *P* = .014) while more were “very satisfied” with their therapy (55% vs 32%, *P* = .002) and a total of 83% reported being “satisfied” or “very satisfied” with their therapy. Overall, 35% of 10 kHz SCS subjects decreased or ceased their opioid consumption. Furthermore, none of the 10 kHz SCS group reported stimulation‐related paresthesia or discomfort while almost half of LF‐SCS subjects reported uncomfortable stimulation.

More detailed 12‐month secondary outcomes published separately demonstrated that 10 kHz SCS was also superior in improving QOL and functional outcomes.^50^ On the Oswestry Disability Index (ODI), 10 kHz SCS subjects reported a greater improvement (difference in medians [DIM]: 6.0 percentage points, *P* = .016) as well as a more favorable distribution among the disability subcategories (*P* = .01). In addition, more of this group moved into a lower disability category (70% vs 55%). They also reported greater improvements in Global Assessment of Functioning (GAF) (DIM: 5.0 points, *P* < .01) as well as in continuous, intermittent, and neuropathic pain (DIM: 1.17, *P* < .005; DIM: 1.33, *P* < .005; and DIM: 0.83, *P* < .01, respectively) measured using the Short‐Form McGill Pain Questionnaire (SF‐MPQ‐2). More of this group were rated as “better” or “a great deal better” on the Clinician Global Impression of Change (CGIC) scale (75% vs 56%, *P* = .009) while the increase in the number classified in the “good sleeper” category on the global Pittsburgh Sleep Quality Index (PSQI) was also higher (*P* = .001). Of those who answered general survey questions, a higher proportion of 10 kHz SCS subjects indicated sleeping and driving with their devices switched on (sleeping: 95% vs 60%, *P* < .001; driving: 94% vs 66%, *P* < .001).

Follow‐up was extended for a year to evaluate 24‐month outcomes.^51^ Data were available for 85 and 71 subjects in the 10 kHz SCS and LF‐SCS groups, respectively. Responder rates for both back pain and leg pain remained statistically superior among the former group (back pain: 76% vs 49%, *P* < .001 for both noninferiority and superiority; leg pain: 73% vs 49%, *P* < .001 for noninferiority and *P* = .003 for superiority). The decrease in both back pain and leg pain was sustained in both groups and remained greater among 10 kHz SCS subjects (back pain: −5.0 vs −3.2 cm, *P* < .001 for both noninferiority and superiority; leg pain: −4.7 vs −3.7 cm, *P* < .001 for noninferiority and *P* = .03 for superiority). Secondary outcomes also reflected the long‐term benefits of 10 kHz SCS with more of this group reporting minimal disability on the ODI (23% vs 10%). Greater numbers were also rated by both clinicians and patients as “a great deal better” on their respective GIC scales (CGIC: 41% vs 20%; PGIC: 34% vs 21%) and reported being “very satisfied” with their therapy (60% vs 40%). The distribution among categories for the ODI, CGIC, and PGIC scales favored 10 kHz SCS (ODI: *P* = .02; CGIC: *P* = .002; PGIC: *P* = .004). A smaller cohort with available data also indicated at 24 months that more LF‐SCS subjects used their device programmer daily (35% vs 0%) and carried it around away from home (85% vs 38%).^50^ Increased reliance on the device programmer may have arisen from uncomfortable paresthesia or discomfort felt during postural changes experienced by 11% and 40%, respectively, of the LF‐SCS subjects who experienced paresthesia (95.5%).

The reported limitations of the study included heterogeneity of pain diagnoses within the subject population. Such diversity in etiology is typically found within the chronic back and leg pain indication. In addition, the study protocol allowed pain medication changes after stimulation activation, although increase in opioid medication was considered as SCS treatment failure. A further limitation was the lack of treatment allocation blinding to the investigator and subject due to the necessity of paresthesia in the LF‐SCS group.

#### 3.1.2 Prospective, multicenter, single‐arm studies

Two prospective, multicenter, single‐arm studies evaluated the benefits of 10 kHz SCS in subjects with a primary diagnosis of chronic back pain. In the first study, six‐month data were presented by Van Buyten et al.^52^ Eighty‐two of 83 enrolled subjects completed a trial, and 72 (88%) achieved sufficient pain relief to receive a permanent system. Within the implanted group, 79% had a diagnosis of FBSS while the remainder had no history of spinal surgery. Response was defined as ≥50% reduction in VAS pain score. At six months postimplantation, 74% and 86% of subjects were back pain and leg pain responders, respectively, while baseline back pain and leg pain decreased by a median of 78% (−5.7 cm, *P* < .001) and 83% (−4.0 cm, *P* < .001), respectively. Notably, 47% of subjects experienced >80% back pain relief. Quality of life measures indicated that disability improved (ODI: 55% to 37%, *P* < .001) along with the rate of sleep disturbances per night (3.7 to 1.3, *P* < .001). At baseline, 86% of subjects were using opioids. By 6 months, 62% of this group had reduced their consumption, and 38% ceased intake. Overall, 85% of subjects were satisfied with their therapy.

At 24 months, results for 65 subjects were reported by Al‐Kaisy et al.^53^ Response rates for back pain and leg pain remained high (60% and 71%, respectively, Figure 1) while the decrease in baseline back pain and leg pain was sustained (back pain: −5.1 cm, *P* < .001; leg pain: −3.1 cm, *P* < .001). The observed improvements from baseline in disability and rate of sleep disturbances were also maintained (ODI: 55% to 40%, *P* < .001; sleep disturbances: 3.7 to 1.4/night, *P* < .001). Furthermore, a smaller proportion of subjects were classified as “crippled” or “severely disabled” (ODI: 90% to 49%). The proportion of subjects using opioids reduced from 86% at baseline to 57% (*P* < .001) while consumption decreased by 68% (MEDD: 84 to 27 mg/d, *P* < .001). Most subjects remained satisfied with their therapy (>80%). Also of note was the comparable level of pain relief found among 15 subjects with no history of spinal surgery (back pain: −4.7 cm, *P* < .001; leg pain: −3.1 cm, *P* < .05).

**Figure 2:**
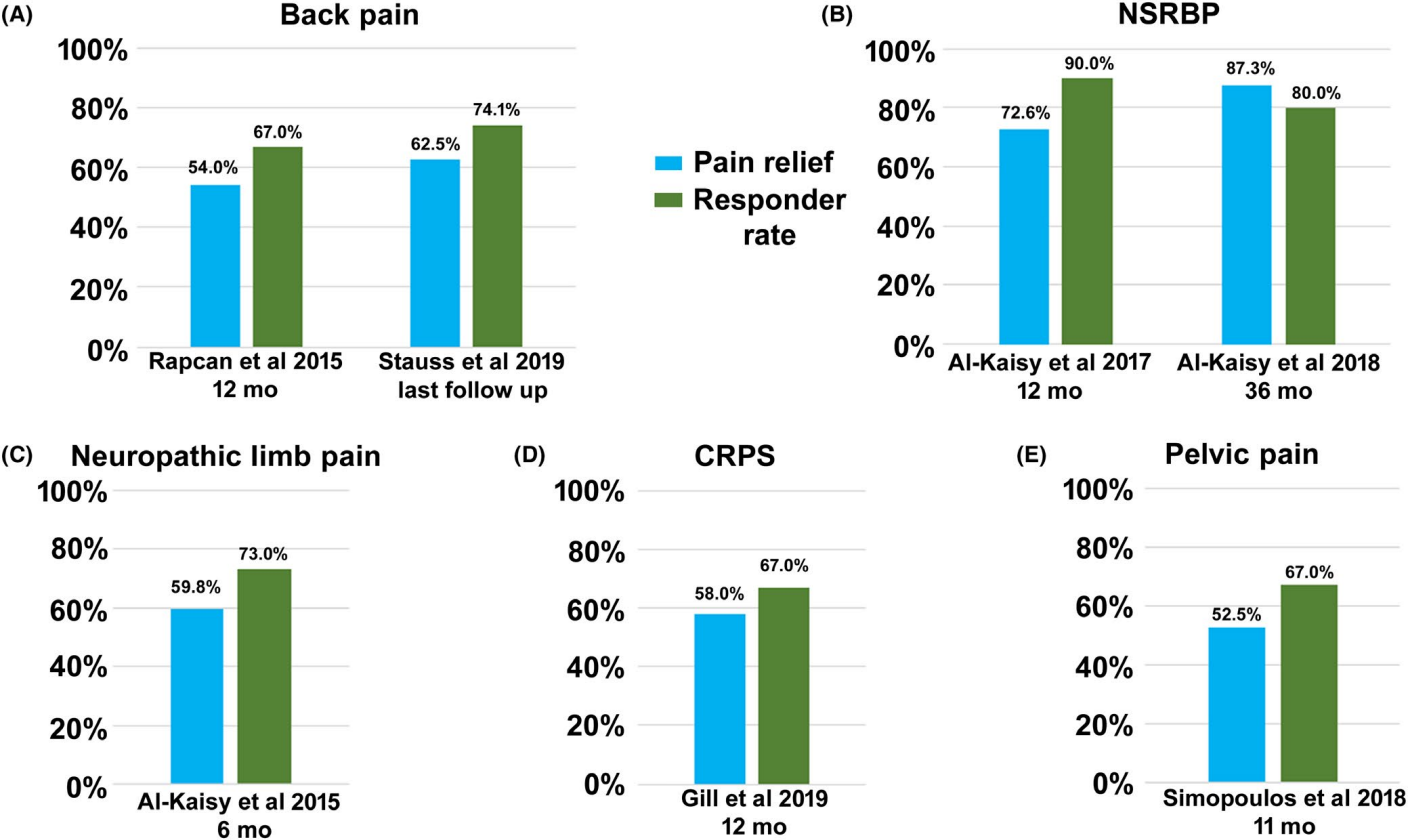
Responder rate and mean pain relief in back pain (A), nonsurgical refractory back pain (B), neuropathic limb pain (C), CRPS (D), and pelvic pain (E) patients

In the second study presented by Rapcan et al^54^, all 21 recruited subjects diagnosed with FBSS completed a successful trial and proceeded to permanent implantation. Pain relief outcomes were collected up to 12 months postimplantation in all subjects. At 12 months, response was observed in 67% of subjects (Figure 2A) while baseline pain decreased by 54% (−4.7 cm, *P* < .001). At the same time point, 65% of the cohort had reduced their opioid consumption by half (Table 2), and performance status (PS) had improved (3.0 to 1.8 points, *P* < .001).

**Table 2:** Studies reporting changes in opioid medication following 10 kHz SCS treatment

References	N	Baseline dose (mg/day)	Last follow‐up dose (mg/day)	% of patients who reduced/eliminated at last follow–up
Kapural et al (2015)^33^	89	112.7	87.9	35.5%
Al‐Kaisy et al (2014)^53^	65	84.0	27.0	72.0%
Al‐Kaisy et al (2017)^57^	20	112.0	40.0	88.0%
DiBenedetto et al (2018)^56^	21	92.2	66.0	71.4%
Stauss et al (2019)^55^	1070	Not reported	Not reported	32.1%
Rapcan et al (2015)^54^	21	Not reported	Not reported	65.0%
Salmon (2019)^64^	24	All patients on opioids: 165.4 Patients on high dose opioids: 210.5	All patients on opioids: 99.3 Patients on high dose opioids: 111.8	79.0%
Arcioni et al (2016)^66^	14	Not reported	Not reported	50.0%
Lambru et al (2016)^68^	4	Not reported	Not reported	100.0%
Gill et al (2019)^60^	3	Not reported	Not reported	33.3%

While a significant strength of the two studies discussed above is their prospective design and length of follow‐up, both were open‐label and lacked a parallel control arm. The level of evidence provided by such studies is much less than an RCT due to the stronger potential for bias and confounding factors. Furthermore, the small sample size in the study reported by Rapcan et al may have introduced additional bias.

#### 3.1.3 Retrospective, real‐world studies

Two retrospective, real‐world studies reported the benefits of 10 kHz SCS in chronic low back and leg pain patients in a clinical setting. In the larger of the two studies, Stauss and associates examined the records of 1660 patients with chronic back and leg pain who were trialed and/or permanently implanted with a 10 kHz SCS system between April 2014 and January 2018 in eight centers.^55^ Throughout the 12‐month study period, approximately 75% of patients with available data reported ≥50% pain relief, corroborated by the last visit evaluation (74%, N = 1131) (Figure 2). In addition, around a third of those with available data noted decreased medication intake while a high proportion indicated improved function (72%), sleep (68%), and quality of life (90%). A considerable strength of this study was its large size and real‐world setting, reflecting everyday clinical practice across several countries. As such, the study provided complementary evidence to the SENZA‐RCT. However, data were collected retrospectively, may not have been collected systematically, and analyses were performed as‐observed.

The smaller of the two studies was conducted in a single center. DiBenedetto and associates compared opioid consumption and procedural volume in 32 patients receiving 10 kHz SCS plus conventional medical management (CMM) with 64 case‐matched controls receiving only CMM.^56^ The study found a decrease in MEDD from baseline to 12 months only among 10 kHz SCS + CMM patients (92.2 to 66.0 mg/d, *P* = .001, N = 21). Although both groups underwent fewer interventional procedures during the 12‐month post‐ vs prebaseline period, a greater decrease was observed among 10 kHz SCS + CMM patients (72% vs 35%, *P* = .03). Furthermore, among 10 kHz SCS + CMM patients, analysis of numerical rating scale (NRS) pain scores at baseline and 12 months revealed a decrease of 46% in low back pain and 51% in lower extremity pain (low back: −3.1 points, *P* < .001, N = 30; lower extremity: −2.9 points, *P* = .01, N = 16). The real‐world nature of this study once again reflects usual clinical practice. However, the retrospective design of the study and small sample size are potentially limiting factors, as is the possibility of heterogeneous baseline characteristics between the treated and untreated patients.

### 3.2 Chronic back pain ineligible for spinal surgery (maiden back or nonsurgical back pain)

The benefits of 10 kHz SCS therapy for subjects with chronic back pain ineligible for spinal surgery and no history of such intervention were evaluated in a separate, single‐center, prospective study by Al‐Kaisy and colleagues.^57^ Twenty of 21 enrolled subjects (95%) had a successful trial, received an implantable pulse generator, and completed 12 months of follow‐up. At 6 and 12 months, 75% and 90% of the cohort were back pain responders (Figure 2B), respectively, while baseline back pain decreased by 60% (−4.7 cm, *P* < .0001) and 73% (−5.6 cm, *P* < .0001), respectively. Even from a low baseline value, leg pain was lower at all time points. At 12 months, disability score almost halved (ODI: −26.0 percentage points, *P* < .0001) while QOL score improved fourfold in the EuroQol 5‐Dimensional Questionnaire Time Trade‐off (EQ5D TTO) valuation (0.16‐0.65 points, *P* < .0001). Both self‐reported subscales of the 36‐Item Short‐Form Health Survey (SF‐36) were also noted to improve (physical component subscale [PCS]: *P* < .0005; mental component subscale [MCS]: *P* < .05). Subjects further reported 54% fewer sleep disturbances (*P* < .05), 64% reduced opioid consumption (MEDD: 112 to 40 mg/d), and at least 70% were satisfied with their therapy (N = 20). Three subjects were able to stop opioid use.

The study investigators extended follow‐up for an additional 24 months and reported findings from the 36‐month assessment.^58^ At 36 months, 80% of subjects had ≥50% reduction in back pain intensity scores, and the average reduction in back pain intensity was 87%. ODI scores reduced from 53.0% at baseline to 19.8% at 36 months (*P* < .0001), and 50% (N = 10/20) of subjects were in the “minimal disability” category. Subjects also continued to wean off their opioid medication: 88% (N = 15/17) of subjects were not taking any opioid medication compared with 10% (N = 17/20) at baseline. EQ5D TTO further improved to 0.84, SF‐36 PCS improved to 48.2, and SF‐36 MCS to 56.8 (*P* < .0001) at the 36‐month assessment.

The study was designed as an exploratory evaluation of the benefits of 10 kHz SCS therapy in this difficult‐to‐treat patient population. A key strength of the study is its observed continued therapeutic benefit after such a long follow‐up period. However, its small number of treated patients, single‐center setting, and single‐arm design are limiting factors.

### 3.3 Neuropathic limb pain

Al‐Kaisy et al^59^ reported the effect of 10 kHz SCS on neuropathic pain in the extremities in a single‐center, retrospective case series. Fifteen enrolled patients had a variety of neuropathic pain syndromes including upper or lower neuropathic limb pain, complex regional pain syndrome (CRPS) of the hand or foot, or postsurgical knee pain. Of these, 11 (73%) had a successful trial, proceeded to permanent implantation, and completed 6 months of follow‐up. At this time point, 73% of the cohort experienced ≥50% reduction in pain score (Figure 2C), with a mean reduction of 59% (−4.9 points, *P* < .05). Improved QOL was observed in a valuation of EQ5D TTO; the score doubled after six months. In addition, patients’ catastrophic thinking related to their pain was markedly reduced over the same period. Most patients reported being satisfied with their therapy (91%). Overall, the study provided insight into the potential benefits of 10 kHz SCS treatment among this diverse patient population. However, the sample size was small, and data were collected retrospectively.

### 3.4 Complex regional pain syndrome

Outcomes of 10 kHz SCS treatment for an exclusive series of CRPS patients were presented in a single‐center, retrospective review by Gill et al.^60^ Eleven of 13 patients (85%) with uni‐ or bilateral CRPS in their upper or lower limbs had a successful trial and received a full system. Despite failing their trial in regard to pain relief, one additional patient was implanted due to vastly improved allodynia. Pain relief (%) was reported during each clinic visit. At a mean follow‐up of 12 months, 67% of patients were responders (Figure 2D). Among the responders were five of seven patients with sympathetically mediated pain (SMP), three of five with sympathetically independent pain (SIP), and five of seven who had previously undergone failed LF‐SCS. Patients also reported significant improvement in all four SF‐MPQ‐2 pain descriptors (continuous, intermittent, neuropathic, and affective: *P* < .01 for all descriptors). While providing preliminary evidence of therapeutic benefit in this challenging pain syndrome, limitations of this study include its small number of patients and retrospective analysis.

### 3.5 LF‐SCS nonresponders

Three studies presented outcomes of 10 kHz SCS treatment in subgroups of LF‐SCS nonresponders. Russo and colleagues retrospectively reviewed 256 patients from three centers who were either not candidates for LF‐SCS or were nonresponders.^61^ Almost half reported both chronic back and leg pain while at least 30% were known to have previously undergone failed LF‐SCS and/or peripheral field nerve stimulation (PNFS). Of the 256 enrolled patients and 76 LF‐SCS/PNFS nonresponders, trial success was reported in 73% and 68% of patients, respectively (all of whom were fully implanted). At 6 months, both groups reported around a 50% reduction in baseline NRS pain score (all patients: −3.8 points, *P* < .001, N = 125; LF‐SCS/PNFS nonresponders: −3.5 points, *P* < .001, N = 38). Among the LF‐SCS/PNFS nonresponders, 55% experienced ≥50% pain relief, and 8% experienced ≥80% pain relief with 10 kHz SCS. Improved disability was reported among the full cohort (ODI: 41.4% to 32.8%, *P* < .001, N = 68). The improvement in ODI score was positively correlated with pain score.

The study by Stauss et al^55^ outlined above also presented pain relief outcomes for a subgroup of patients who had undergone previously failed LF‐SCS. Overall, the subgroup baseline characteristics, trial results, and pain relief outcomes mirrored those of the entire cohort. The responder rate was 76% at 3 months (N = 193), sustained through 12 months (79%, N = 90), and corroborated by the last visit evaluation (74%, N = 266). Also similar to the main group was the proportion of patients with available data indicating decreased medication intake (32%), improved function (82%) and sleep (70%), better quality of life (88%), satisfaction with therapy (≥80%), and driving/sleeping with their device switched on (98%).

The CRPS study by Gill et al discussed above included seven patients who failed previous LF‐SCS treatment.^60^ After a successful response during trial stimulation, patients decided to replace their old device with 10 kHz SCS during the insertion phase. At last follow‐up, five out of seven patients (71%) achieved ≥50% pain relief, and the remaining two achieved the minimum clinically important change (30% pain relief).^62^, ^63^

While subgroup analyses can help identify and optimize new therapeutic applications, they present many analytic challenges and pitfalls. As such, while the subgroup data for LF‐SCS nonresponders presented above is certainly promising, it should be interpreted with caution.

### 3.6 Chronic widespread pain (off‐label indication for SCS)

A recent, single‐center, retrospective review by Salmon et al documented long‐term outcomes of 10 kHz SCS in patients with combined upper and lower body neuropathic/nociplastic pain syndromes.^64^ Thirty‐eight of 45 patients (84%) had a successful trial and received a full system. Ten implanted patients had previously undergone failed LF‐SCS and/or PNFS. Mean follow‐time was 2.3 years. Last follow‐up data were analyzed for 35 patients (92%) who were still using their implanted system for pain management. This group reported an average decrease of 48% in baseline NRS pain score (−3.5 points, *P* = .00001). Pain relief was approximately 60% in the head and neck region, as well as in the upper and lower back areas. Only two of 35 patients reported pain relief ≤40%. Improved disability was found among those who completed the Roland Morris Disability Questionnaire (RMDQ: 12.3 to 7.8 points, *P* ≤ .05, N = 29). The number of patients taking strong opioids decreased from 24 at baseline to 15. Among the 15 patients who continued opioid therapy, consumption reduced by 40% (MEDD: 165.4 to 99.3 mg/day). Of these, 11 were taking high doses of opioids at baseline. This cohort reduced their consumption by 47% (MEDD: 210.5 to 111.8 mg/day, *P* < .05). Of the 29 patients with available data, more than three‐quarters reported themselves “moderate to a great deal better” on the PGIC while most were satisfied with their therapy (93%). Also of note was the increase in the proportion of work‐eligible patients who were employed at the end of follow‐up (26% to 64%, N = 31). Patients who returned to work attributed this change primarily to the pain relief resulting from 10 kHz SCS therapy. Particular strengths of this study were its long average follow‐up period and the number of enrolled patients. However, the retrospective design of the study is a disadvantage.

### 3.7 Chronic pelvic pain

Simopoulos and associates presented three chronic pelvic pain case studies treated with 10 kHz SCS.^65^ The first patient had severe, unilateral, coccydynia pain (without radiation) after coccygectomy. He reported pain intensity of 8.2 cm (VAS) and a sitting tolerance of 15 minutes. Previously failed therapies included pharmacotherapy as well as radiofrequency and cryoablation. During a 10 kHz SCS trial, the patient experienced a 50% reduction in pain. Nine months after permanent implantation, the patients reported a reduction in baseline pain intensity of 51% (VAS: −4.2 cm) and an eightfold improvement in sitting tolerance (15 to 120 min).

The second patient reported rectal pain with associated burning and numbness in the scrotum and penis following a trauma‐induced cauda equina syndrome. After an extensive laminectomy from L1 to L5, the function of his legs recovered. However, numerous attempts to provide adequate pain relief, including pharmacotherapy, physical therapy, nerve blocks, and anterograde LF‐SCS, were unsuccessful. Low‐frequency sacral nerve root stimulation initially provided 50% pain relief. However, the benefit diminished during the year after permanent implantation. A subsequent trial of 10 kHz SCS provided 60% pain relief. Twelve months after full implantation, the patient's level of pain relief from baseline was maintained (VAS: 3.3 cm), accompanied by a 75% reduction in opioids.

The final patient had pudendal neuralgia presenting as pain in the vagina, rectum, and coccyx, which was aggravated by sitting. After exhausting numerous treatments, including pharmacotherapy and physical therapy as well as pudendal nerve blocks, decompression surgeries, and radiofrequency lesioning, her average pain intensity was 7.5 cm (VAS). A trial of 10 kHz SCS resulted in complete resolution of her pain with no sitting‐related aggravation. Eleven months after permanent implantation, the patient reported a reduction in baseline pain of 45% (VAS: 4.1 cm).

These cases suggest that 10 kHz SCS therapy can provide relief for chronic visceral pain (Figure 2E). However, the number of patients was small and the follow‐up time was short. Further studies are warranted to define the place of SCS and 10 kHz SCS in visceral pain syndromes.

### 3.8 Intractable headache (off‐label indication for SCS)

Another possible indication for 10 kHz SCS to date beyond trunk and/or limb pain is intractable headache (HA). A prospective, open‐label study by Arcioni and associates trialed the therapy in 17 subjects with refractory chronic migraine (CM).^66^ Of these, 15 elected to have a permanent system, and 14 completed six months of follow‐up. All subjects had failed botulinum toxin therapy and were overusing medication at baseline. Continuation of usual medication was allowed throughout the study, including migraine preventatives (although none took any of the latter). The definition of a HA day was defined according to standard criteria (>4 hours of continuous HA, either with NRS > 4, or, if taking abortive medication, NRS > 0).^67^ At six months, half of the cohort experienced more than 30% fewer monthly HA days, and 36% had more than 50% fewer monthly HA days, with an average reduction for the whole group of 7.0 days (*P* = .004). More than half of the subjects reverted to an episodic migraine pattern. Average HA intensity was noted to decrease by 37% (*P* < .001) while half of the cohort experienced at least a 30% reduction (mean reduction: −3.3 points). The total monthly number of HA hours reduced by 17% (*P* = .05) while 43% of subjects reported more than a 30% reduction (mean reduction: 92 hours). Evaluation of HA‐related disability and functional ability according to the Migraine Disability Assessment Scale (MIDAS) and Headache Impact Test (HIT‐6) revealed concomitant improvements in overall score (MIDAS: −115 points, *P* < .001; HIT‐6: −8.3 points, *P* < .01) as well as in the proportion of subjects reporting severe disability (MIDAS: 100% to 69%; HIT‐6: 100% to 62%). Moreover, the percentage of subjects overusing triptans or other analgesics reduced substantially (triptans: 64% to 36%; other analgesics: 36% to 14%), and four subjects were able to discontinue triptan use. While the study was prospective in design, the objective of the study was exploratory. The sample size was therefore small. Further studies are recommended to evaluate the safety and efficacy of the therapy in larger CM populations.

In a retrospective case series, Lambru et al reported the efficacy of 10 kHz SCS for the treatment of various refractory primary HA types, including CM, chronic short‐lasting unilateral neuralgiform headache attacks with autonomic symptoms (SUNA), and chronic cluster headache (CCH). Seven patients completed successful trials prior to full implant.^68^ All four CM patients were overusing medication at baseline, three underwent medication withdrawal, three had tried and failed botulinum toxin therapy, and one had failed traditional low‐frequency occipital nerve stimulation (LF‐ONS). The standard definitions of HA and migraine days were used.^67^ At 25 months average follow‐up, all CM patients reported ≥50% reduction in monthly HA and migraine days as well as reversion to an episodic migraine pattern. Two of the four CM patients experienced a “meaningful” improvement in disability (HIT‐6) while three reduced their analgesic consumption, and one who took daily sumatriptan reduced their intake to 1 day per month. Headache improvement occurred just a few days after trial stimulation in most CM patients (3/4), and all rated their overall HA improvement as between 50% and 100%. One of the two SUNA patients had remission of her frequent attacks (30‐50/day, 2‐600 s duration, VRS 8‐10 points) for eight months after implantation before her attacks returned. However, both the frequency, duration, and intensity were less at 28 months (10‐20/day, 2‐30 s duration, VRS 5‐7 points). She rated her overall improvement as 70%, stopped taking all preventative medication since implant, and managed to return to work full‐time. The other SUNA patient experienced a dramatic improvement in attack frequency in the 16 months after implant (50‐60/day to 2/month) as well as a reduction in intensity (VRS: 10 to 6 points). At their last follow‐up (42 months from implant), the patient reported almost complete resolution of SUNA attacks as well as the CM, and no adverse effects. The patient with CCH had previously undergone LF‐ONS with remission of his HA but relapsed after six months. After implantation with a 10 kHz SCS system, he was HA free for 9 months. Subsequently, his attacks gradually returned to the same frequency and intensity, but the duration of attack was significantly reduced (40‐180 minutes at baseline to 20‐35 minutes at the last follow‐up reported); overall improvement rated by the patient was 50%. In these medically refractory primary chronic HA patients, 10 kHz SCS treatment was beneficial. However, the number of included patients was small, and data were collected retrospectively.

### 3.9 Ongoing studies evaluating 10 kHz SCS therapy

The utility of the therapy in treating other challenging pain syndromes such as upper limb and neck pain, polyneuropathy including painful diabetic neuropathy (PDN), chronic postsurgical pain, chronic pelvic pain, and nonsurgical back pain is currently being explored in several multicenter, prospective, single‐arm studies or RCTs. The complete/interim results and the trial designs of the studies were presented at the 22nd Annual Meeting of the North American Neuromodulation Society (NANS), January 17‐20, 2019, Las Vegas, NV.

#### 3.9.1 Chronic intractable upper limb and/or neck pain (neck pain is off‐label indication for SCS in the US)

Amirdelfan and colleagues presented complete 12‐month data from a single‐arm study of chronic upper limb and/or neck pain (NCT02385201).^69^, ^70^ The most commonly reported pain etiologies were radiculopathy/neuropathic pain (89%), degenerative disk disease (71%), and failed cervical spine surgery syndrome (56%). Of the 55 enrolled subjects, 76% reported neck pain, 44% reported upper limb pain, and 89% completed a successful trial followed by permanent implantation. At 12 months, regardless of reported pain location (upper limb pain: N = 20; neck pain: N = 37), around 90% of subjects were responders (≥50% reduction in VAS pain score) while baseline pain score decreased by about six cm. Subjects further reported reductions of at least 66% in all SF‐MPQ‐2 pain descriptors as well as improved disability on the Pain Disability Index (PDI: 42.4 to 16.9 points, N = 37).

Burgher and associates described therapy outcomes in a series of subjects with chronic upper extremity pain (NCT02703818).^71^ Thirty‐eight of 42 subjects (90%) completed successful trials. Of these, 33 received permanent systems, and 30 completed 12 months of scheduled follow‐up. At 12 months, response (≥50% reduction in VAS pain score) was observed in 73%, 87%, and 80% of subjects with neck, shoulder, and upper limb pain, respectively, while median baseline pain score decreased by at least seven cm, regardless of pain location. Disability improved on both the PDI and Quick Disabilities of Arm, Shoulder & Hand (QuickDASH) scales (median PDI: 49.0 to 15.5 points; median QuickDASH: 70.4 to 31.8 points). Subjects also reported better function (median GAF: 55.0 to 75.0 points) and sleep (median Pain and Sleep Questionnaire three‐item index [PSQ3]: 25.2 to 5.5 points), and most were satisfied with their therapy (87%).

#### 3.9.2 Peripheral polyneuropathy

Galan et al^72^ summarized results from a single cohort of subjects with peripheral polyneuropathy of the upper or lower limbs. Of the 28 enrolled subjects, 26 underwent trials, 21 had successful trials (81%), and 18 received a permanent implant. A 24‐month interim analysis revealed sustained benefit throughout follow‐up. Response (≥50% reduction in VAS pain score) was observed in 87% of subjects (N = 8) at 24 months as well as a decrease in baseline pain score of 6.5 cm. At the same time point, all SF‐MPQ‐2 sensory pain descriptors reduced by at least 50% while the affective pain descriptor decreased by 80%. Disability was also noted to improve (PDI: 40.3 to 12.3 points). In the PDN subgroup (9/26), the response rate was >80% for most of the follow‐up period, but slightly lower at 67% by the end of follow‐up.

#### 3.9.3 Chronic postsurgical pain

In a single‐arm, chronic postsurgical pain study presented by Gupta and colleagues, 29 of 34 subjects (85%) trialed with 10 kHz SCS experienced adequate pain relief and were fully implanted.^73^ The reported 12‐month interim analysis revealed response (≥50% reduction in VAS pain score) in 87% of subjects (20/23) at 12 months as well as a decrease in baseline pain score of 6.5 cm. At the same follow‐up time, a reduction of more than 70% in all SF‐MPQ‐2 pain descriptors was observed along with improved disability (PDI: 42.1 to 12.5 points, N = 20). The lower extremity pain subgroup within this study reported similar pain relief outcomes to the full cohort.

#### 3.9.4 Chronic abdominal pain (off‐label indication for SCS)

Kapural et al^74^ presented outcomes from a series of 24 subjects with chronic abdominal pain. All but one (96%) had a successful trial and received a full system. Analysis of complete 12‐month data demonstrated response (≥50% reduction in VAS pain score) in 78% of the cohort at 12 months with a decrease in baseline pain score of 6.0 cm. Subjects further reported around 70% less sleep disturbance (PSQ3) while around three‐quarters of the cohort reported being better (PGIC). Concomitant improvements in gastrointestinal symptoms were also noted.

#### 3.9.5 Chronic pelvic pain

In a single‐arm study of chronic pelvic pain summarized by Tate et al, 21 subjects underwent a trial.^75^ Of these, 17 (81%) achieved adequate pain relief and were implanted with a permanent system. Follow‐ups were scheduled up to 12 months postimplantation. A 3‐month interim analysis revealed response (≥50% reduction in VAS pain score) in 79% of subjects (11/14) at 3 months, along with a 5.1 cm reduction in baseline pain. At the same time point in 13 subjects, all SF‐MPQ‐2 subscales decreased by around 60% while disability improved (PDI: 43.3 to 18.3 points). Subjects also reported benefits to their sleep. Data were available for only five subjects at 12 months. However, the benefits were sustained across all outcome measures.

#### 3.9.6 Painful diabetic neuropathy

In the first of three RCTs outlined during the NANS conference, subjects with neuropathic limb pain secondary to PDN are being enrolled.^76^ The study will compare 10 kHz SCS treatment plus CMM with CMM alone (NCT03228420). A total of 216 subjects will be randomized (1:1) and followed for 24 months. Outcome measures include pain score, health‐related quality of life (HRQOL), and sleep quality. The primary endpoint will compare group responder rates at 3 months. Enrollment is expected to complete during 2019.

#### 3.9.7 Nonsurgical refractory back pain

The second RCT is currently recruiting subjects with chronic back pain who have not had spinal surgery and are not candidates for such surgery (NCT03680846).^77^ The protocol specifies randomization (1:1) into two treatment groups: 10 kHz SCS plus CMM versus CMM alone. Subjects can cross over at 6 months and will be followed out to 12 months. Outcome measures include pain score, HRQOL, sleep, GIC, mental health, disability, opioid consumption, and healthcare utilization. The primary endpoint will compare group responder rates at 12 months. Enrollment is expected to continue until the end of 2019.

#### 3.9.8 Chronic neuropathic low back pain

The third RCT is also underway and enrolling subjects with chronic neuropathic low back pain who are surgery naïve (NCT03470766).^78^ The double‐blind, multicenter study will compare active 10 kHz SCS plus usual care with sham 10 kHz SCS plus usual care. A total of 96 subjects will be randomized 1:1 and followed for six months. Outcome measures include pain score, disability, emotional functioning, HRQOL, medication usage, and healthcare utilization. Enrollment is expected to complete during 2020.

## 4 SUMMARY

The primary aim of this review was to summarize the current clinical evidence for the use of 10 kHz SCS in the treatment of various chronic pain conditions. Several studies provide significant and converging evidence that this therapy is a clinically effective treatment for chronic back and leg pain, including an RCT as well as prospective and retrospective studies. Level I evidence was established by a pivotal, multicenter, RCT, which compared 10 kHz SCS with LF‐SCS.^33^, ^51^ The study found long‐term statistically superior pain relief among those treated with 10 kHz SCS. Two prospective, single‐arm studies found similarly high levels of response to therapy, and two retrospective studies confirmed that 10 kHz SCS is effective in real‐world settings.^52^, ^53^, ^54^, ^55^, ^56^ Among the five studies, pain relief outcomes from >1000 subjects were evaluated at the end of follow‐up (12‐24 months). Twelve‐month responder rates exceeded 70%, and 24‐month responder rates ranged from 60% to 80%. Most of the studies reported quality of life and disability improvements as well as a reduction in opioid consumption. In general, at least 80% of subjects reported being satisfied with their therapy.

The results from studies and case series evaluating therapy outcomes in other indications, including chronic back pain ineligible for spinal surgery, neuropathic limb pain, CRPS, chronic widespread pain, chronic pelvic pain, and intractable headache, are promising. In subjects with chronic, severe, low back pain who were not candidates for spinal surgery and were naïve to surgery, the 12‐month response rate in the prospective study by Al‐Kaisy and colleagues was strikingly high.^57^ The results suggest that 10 kHz SCS may be a viable option in this population where treatment possibilities are limited. Promising reductions in pain were also found in retrospective studies of neuropathic limb pain, chronic widespread pain, and CRPS.^59^, ^60^, ^64^ Given the difficulty in managing these pain syndromes and their particularly devastating impact on patients’ lives, this certainly merits further exploration, which is currently being undertaken in the form of multicenter, randomized controlled trials. Subgroup analyses also point toward the potential of 10 kHz SCS being successful even when LF‐SCS has failed.^55^, ^61^ Individually, the studies provide preliminary evidence supporting the use of 10 kHz SCS in a wide variety of pain conditions. Larger confirmatory studies are necessary, and multiple randomized, controlled trials in these pain conditions are currently underway.

Our narrative review has several key limitations. Firstly, it was not designed as a formal systematic review. Furthermore, prospective case series published in peer‐reviewed journals were single‐arm in design while prospective data from ongoing studies were reported during conference proceedings. The evidence level provided by other case series is also limited by their retrospective design and, in many cases, small sample size. However, their findings may inform the implementation and design of future RCTs similar to the SENZA‐RCT. The review also includes off‐label applications of the therapy. Authors of this article do not recommend the use of SCS therapy for off‐label applications in the US until stronger evidence and/or FDA approval is available for these indications, including intractable headache, chronic intractable neck pain, and chronic abdominal pain.

In conclusion, 10 kHz SCS has been shown to provide long‐term pain relief in various chronic pain etiologies. The magnitude of the relief shown has been superior to previous studies and real‐world data on low‐frequency stimulation. This relief has also been associated with improved quality of life and reduced opioid consumption. Ongoing and future research will continue to investigate the therapy in current and new indications, and the findings will be summarized in upcoming publications.

## CONFLICTS OF INTEREST

Dr Dawood Sayed, Dr Jan Willem Kallewaard, and Dr Jessica Jameson are consultants of Nevro Corp. Dr David Caraway and Dr Anand Rotte are employees of Nevro Corp.

## References

[1] Dahlhamer J , Lucas J , Zelaya C , et al. Prevalence of chronic pain and high‐Impact chronic pain among adults ‐ United States, 2016. MMWR Morb Mortal Wkly Rep. 2018;67(36):1001‐1006. 30212442 10.15585/mmwr.mm6736a2 PMC6146950

[2] Disease GBD , Injury I , Prevalence C . Global, regional, and national incidence, prevalence, and years lived with disability for 354 diseases and injuries for 195 countries and territories, 1990–2017: a systematic analysis for the Global Burden of Disease Study 2017. Lancet. 2018;392(10159):1789‐1858. 30496104 10.1016/S0140-6736(18)32279-7 PMC6227754

[3] Breivik H , Collett B , Ventafridda V , Cohen R , Gallacher D . Survey of chronic pain in Europe: prevalence, impact on daily life, and treatment. Eur J Pain. 2006;10(4):287‐333. 16095934 10.1016/j.ejpain.2005.06.009

[4] Pitcher MH , Von Korff M , Bushnell MC , Porter L . Prevalence and profile of high‐impact chronic pain in the United States. J Pain. 2019;20(2):146‐160. 30096445 10.1016/j.jpain.2018.07.006 PMC8822465

[5] Fayaz A , Croft P , Langford RM , Donaldson LJ , Jones GT . Prevalence of chronic pain in the UK: a systematic review and meta‐analysis of population studies. BMJ Open. 2016;6(6):e010364. 10.1136/bmjopen-2015-010364 PMC4932255 27324708

[6] Johannes CB , Le TK , Zhou X , Johnston JA , Dworkin RH . The prevalence of chronic pain in United States adults: results of an Internet‐based survey. J Pain. 2010;11(11):1230‐1239. 20797916 10.1016/j.jpain.2010.07.002

[7] Eriksen J , Jensen MK , Sjøgren P , Ekholm O , Rasmussen NK . Epidemiology of chronic non‐malignant pain in Denmark. Pain. 2003;106(3):221‐228. 14659505 10.1016/S0304-3959(03)00225-2

[8] Elliott AM , Smith BH , Penny KI , Cairns Smith W , Alastair CW . The epidemiology of chronic pain in the community. Lancet. 1999;354(9186):1248‐1252. 10520633 10.1016/s0140-6736(99)03057-3

[9] Freburger JK , Holmes GM , Agans RP , et al. The rising prevalence of chronic low back pain. Arch Intern Med. 2009;169(3):251‐258. 19204216 10.1001/archinternmed.2008.543 PMC4339077

[10] Palmer KT . Back pain in Britain: comparison of two prevalence surveys at an interval of 10 years. BMJ. 2000;320(7249):1577‐1578. 10845966 10.1136/bmj.320.7249.1577 PMC27402

[11] Tsang A , Von Korff M , Lee S , et al. Common chronic pain conditions in developed and developing countries: gender and age differences and comorbidity with depression‐anxiety disorders. J Pain. 2008;9(10):883‐891. 18602869 10.1016/j.jpain.2008.05.005

[12] Leadley RM , Armstrong N , Reid KJ , Allen A , Misso KV , Kleijnen J . Healthy aging in relation to chronic pain and quality of life in Europe. Pain Pract. 2014;14(6):547‐558. 24138082 10.1111/papr.12125

[13] Mathias JL , Cant ML , Burke ALJ . Sleep disturbances and sleep disorders in adults living with chronic pain: a meta‐analysis. Sleep Med. 2018;52:198‐210. 30314881 10.1016/j.sleep.2018.05.023

[14] Bair MJ , Robinson RL , Katon W , Kroenke K . Depression and pain comorbidity: a literature review. Arch Intern Med. 2003;163(20):2433‐2445. 14609780 10.1001/archinte.163.20.2433

[15] Rayner L , Hotopf M , Petkova H , Matcham F , Simpson A , McCracken LM . Depression in patients with chronic pain attending a specialised pain treatment centre: prevalence and impact on health care costs. Pain. 2016;157(7):1472‐1479. 26963849 10.1097/j.pain.0000000000000542 PMC4912238

[16] Duenas M , Ojeda B , Salazar A , Mico JA , Failde I . A review of chronic pain impact on patients, their social environment and the health care system. J Pain Res. 2016;9:457‐467. 27418853 10.2147/JPR.S105892 PMC4935027

[17] Reid KJ , Harker J , Bala MM , et al. Epidemiology of chronic non‐cancer pain in Europe: narrative review of prevalence, pain treatments and pain impact. Curr Med Res Opin. 2011;27(2):449‐462. 21194394 10.1185/03007995.2010.545813

[18] West C , Usher K , Foster K , Stewart L . Chronic pain and the family: the experience of the partners of people living with chronic pain. J Clin Nurs. 2012;21(23–24):3352‐3360. 22834990 10.1111/j.1365-2702.2012.04215.x

[19] Patel AS , Farquharson R , Carroll D , et al. The impact and burden of chronic pain in the workplace: a qualitative systematic review. Pain Pract. 2012;12(7):578‐589. 22462774 10.1111/j.1533-2500.2012.00547.x

[20] Gaskin DJ , Richard P . The economic costs of pain in the United States. J Pain. 2012;13(8):715‐724. 22607834 10.1016/j.jpain.2012.03.009

[21] Raftery MN , Ryan P , Normand C , Murphy AW , de la Harpe D , McGuire BE . The economic cost of chronic noncancer pain in Ireland: results from the PRIME study, part 2. J Pain. 2012;13(2):139‐145. 22300900 10.1016/j.jpain.2011.10.004

[22] Gustavsson A , Bjorkman J , Ljungcrantz C , et al. Socio‐economic burden of patients with a diagnosis related to chronic pain–register data of 840,000 Swedish patients. Eur J Pain. 2012;16(2):289‐299. 22323381 10.1016/j.ejpain.2011.07.006

[23] Allegri M , Lucioni C , Mazzi S , Serra G . Social cost of chronic pain in Italy. Glob Reg Health Technol Assess. 2015;2:33‐42.

[24] Pain: Assessment, non‐opioid treatment approaches and opioid management (health care guideline). Institute for Clinical Systems Improvement; 2017.

[25] Meske DS , Lawal O , Elder H , Langberg V , Paillard F , Katz N . Efficacy of opioids versus placebo in chronic pain: a systematic review and meta‐analysis of enriched enrollment randomized withdrawal trials. J Pain Res. 2018;11:923‐934. 29765246 10.2147/JPR.S160255 PMC5939920

[26] Chou R , Turner JA , Devine EB , et al. The effectiveness and risks of long‐term opioid therapy for chronic pain: a systematic review for a National Institutes of Health Pathways to Prevention Workshop. Ann Intern Med. 2015;162(4):276‐286. 25581257 10.7326/M14-2559

[27] Stannard CD . Where now for opioids in chronic pain? Drug Ther Bull. 2018;56(10):118‐122. 30254063 10.1136/dtb.2018.10.000007

[28] Boudreau D , Von Korff M , Rutter CM , et al. Trends in long‐term opioid therapy for chronic non‐cancer pain. Pharmacoepidemiol Drug Saf. 2009;18(12):1166‐1175. 19718704 10.1002/pds.1833 PMC3280087

[29] Gharibo C , Laux G , Forzani BR , Sellars C , Kim E , Zou S . State of the field survey: spinal cord stimulator use by academic pain medicine practices. Pain Med. 2014;15(2):188‐195. 24138612 10.1111/pme.12264

[30] Moore DM , McCrory C . Spinal cord stimulation. BJA Educ. 2016;16(8):258‐263.

[31] North RB , Kidd DH , Farrokhi F , Piantadosi SA . Spinal cord stimulation versus repeated lumbosacral spine surgery for chronic pain: a randomized, controlled trial. Neurosurgery. 2005;56(1): 98‐107; discussion 106–107. 10.1227/01.neu.0000144839.65524.e0 15617591

[32] Kumar K , Taylor RS , Jacques L , et al. Spinal cord stimulation versus conventional medical management for neuropathic pain: a multicentre randomised controlled trial in patients with failed back surgery syndrome. Pain. 2007;132(1–2):179‐188. 17845835 10.1016/j.pain.2007.07.028

[33] Kapural L , Yu C , Doust MW , et al. Novel 10‐kHz high‐frequency therapy (HF10 Therapy) is superior to traditional low‐frequency spinal cord stimulation for the treatment of chronic back and leg pain: The SENZA‐RCT randomized controlled trial. Anesthesiology. 2015;123(4):851‐860. 26218762 10.1097/ALN.0000000000000774

[34] Grider JS , Manchikanti L , Carayannopoulos A , et al. Effectiveness of spinal cord stimulation in chronic spinal pain: A systematic review. Pain Physician. 2016;19(1):E33‐54. 26752493

[35] Bala MM , Riemsma RP , Nixon J , Kleijnen J . Systematic review of the (cost‐)effectiveness of spinal cord stimulation for people with failed back surgery syndrome. Clin J Pain. 2008;24(9):741‐756. 18936591 10.1097/AJP.0b013e318179032a

[36] Taylor RS , Ryan J , O'Donnell R , Eldabe S , Kumar K , North RB . The cost‐effectiveness of spinal cord stimulation in the treatment of failed back surgery syndrome. Clin J Pain. 2010;26(6):463‐469. 20551721 10.1097/AJP.0b013e3181daccec

[37] Farber SH , Han JL , Elsamadicy AA , et al. Long‐term cost utility of spinal cord stimulation in patients with failed back surgery syndrome. Pain Physician. 2017;20(6):E797‐E805. 28934786 PMC8358894

[38] Lad SP , Babu R , Bagley JH , et al. Utilization of Spinal Cord Stimulation in Patients With Failed Back Surgery Syndrome. Spine. 2014;39(12):E719‐E727. 24718057 10.1097/BRS.0000000000000320

[39] Linderoth B , Foreman RD . Conventional and novel spinal stimulation Algorithms: Hypothetical Mechanisms of Action and Comments on Outcomes. Neuromodulation. 2017;20(6):525‐533. 28568898 10.1111/ner.12624

[40] Kuechmann C , Valine T , Wolfe DL . 853 Could automatic position adaptive stimulation be useful in spinal cord stimulation? Eur J Pain. 2009;13:S243.

[41] Levy RM . Anatomic considerations for spinal cord stimulation. Neuromodulation. 2014;17(Suppl 1):2‐11. 24974771 10.1111/ner.12175

[42] Schultz DM , Webster L , Kosek P , Dar U , Tan Y , Sun M . Sensor‐driven position‐adaptive spinal cord stimulation for chronic pain. Pain Physician. 2012;15(1):1‐12. 22270733

[43] Kupers RC , Van den Oever R , Van Houdenhove B , et al. Spinal cord stimulation in Belgium: A nation‐wide survey on the incidence, indications and therapeutic efficacy by the health insurer. Pain. 1994;56(2):211‐216. 8008410 10.1016/0304-3959(94)90096-5

[44] Ohnmeiss DD , Rashbaum RF , Bogdanffy GM . Prospective outcome evaluation of spinal cord stimulation in patients with intractable leg pain. Spine. 1996;21(11):1344‐1350; discussion 1351. 8725927 10.1097/00007632-199606010-00013

[45] Alo KM , Redko V , Charnov J . Four year follow‐up of dual electrode spinal cord stimulation for chronic pain. Neuromodulation. 2002;5(2):79‐88. 22151846 10.1046/j.1525-1403.2002.02017.x

[46] Cameron T . Safety and efficacy of spinal cord stimulation for the treatment of chronic pain: a 20‐year literature review. J Neurosurg. 2004;100(3 Suppl Spine):254‐267. 15029914 10.3171/spi.2004.100.3.0254

[47] Sears NC , Machado AG , Nagel SJ , et al. Long‐term outcomes of spinal cord stimulation with paddle leads in the treatment of complex regional pain syndrome and failed back surgery syndrome. Neuromodulation. 2011;14(4): 312‐318; discussion 318. 21992424 10.1111/j.1525-1403.2011.00372.x

[48] Kumar K , Hunter G , Demeria D . Spinal cord stimulation in treatment of chronic benign pain: challenges in treatment planning and present status, a 22‐year experience. Neurosurgery. 2006;58(3):481‐496; discussion 481–496. 16528188 10.1227/01.NEU.0000192162.99567.96

[49] Russo M , Van Buyten JP . 10‐kHz high‐frequency SCS therapy: a clinical summary. Pain Med. 2015;16(5):934‐942. 25377278 10.1111/pme.12617 PMC4660894

[50] Amirdelfan K , Yu C , Doust MW , et al. Long‐term quality of life improvement for chronic intractable back and leg pain patients using spinal cord stimulation: 12‐month results from the SENZA‐RCT. Qual Life Res. 2018;27(8):2035‐2044. 29858746 10.1007/s11136-018-1890-8

[51] Kapural L , Yu C , Doust MW , et al. Comparison of 10‐kHz high‐frequency and traditional low‐frequency spinal cord stimulation for the treatment of chronic back and leg Pain: 24‐Month Results from a multicenter, randomized, controlled pivotal trial. Neurosurgery. 2016;79(5):667‐677. 27584814 10.1227/NEU.0000000000001418 PMC5058646

[52] Van Buyten JP , Al‐Kaisy A , Smet I , Palmisani S , Smith T . High‐frequency spinal cord stimulation for the treatment of chronic back pain patients: results of a prospective multicenter European clinical study. Neuromodulation. 2013;16(1): 59‐66; discussion 65–56. 23199157 10.1111/ner.12006

[53] Al‐Kaisy A , Van Buyten JP , Smet I , Palmisani S , Pang D , Smith T . Sustained effectiveness of 10 kHz high‐frequency spinal cord stimulation for patients with chronic, low back pain: 24‐month results of a prospective multicenter study. Pain Med. 2014;15(3):347‐354. 24308759 10.1111/pme.12294 PMC4282782

[54] Rapcan R , Mlaka J , Venglarcik M , Vinklerova V , Gajdos M , Illes R . High‐frequency – spinal cord stimulation. Bratislava Med J. 2015;116(06):354‐356. 10.4149/bll_2015_067 26084736

[55] Stauss T , El Majdoub F , Sayed D , et al. A multicenter real‐world review of 10 kHz SCS outcomes for treatment of chronic trunk and/or limb pain. Ann Clin Transl Neurol. 2019;6(3):496‐507. 30911573 10.1002/acn3.720 PMC6414485

[56] DiBenedetto DJ , Wawrzyniak KM , Schatman ME , Kulich RJ , Finkelman M . 10 kHz spinal cord stimulation: a retrospective analysis of real‐world data from a community‐based, interdisciplinary pain facility. J Pain Res. 2018;11:2929‐2941. 30538532 10.2147/JPR.S188795 PMC6251433

[57] Al‐Kaisy A , Palmisani S , Smith TE , et al. 10 kHz High‐frequency spinal cord stimulation for chronic axial low back pain in patients with no history of spinal surgery: A preliminary, prospective, open label and proof‐of‐concept study. Neuromodulation. 2017;20(1):63‐70. 28025843 10.1111/ner.12563

[58] Al‐Kaisy A , Palmisani S , Smith TE , et al. Long‐term improvements in chronic axial low back pain patients without previous spinal surgery: A cohort analysis of 10‐kHz high‐frequency spinal cord stimulation over 36 months. Pain Med. 2018;19(6):1219‐1226. 29077889 10.1093/pm/pnx237

[59] Al‐Kaisy A , Palmisani S , Smith T , Harris S , Pang D . The use of 10‐kilohertz spinal cord stimulation in a cohort of patients with chronic neuropathic limb pain refractory to medical management. Neuromodulation. 2015;18(1):18‐23; discussion 23. 25257382 10.1111/ner.12237

[60] Gill JS , Asgerally A , Simopoulos TT . High‐frequency spinal cord stimulation at 10 kHz for the treatment of complex regional pain syndrome: A case Series of patients with or without previous spinal cord stimulator implantation. Pain Pract. 2019;19(3):289‐294. 30365222 10.1111/papr.12739

[61] Russo M , Verrills P , Mitchell B , Salmon J , Barnard A , Santarelli D . High frequency spinal cord stimulation at 10 kHz for the treatment of chronic pain: 6‐Month Australian clinical experience. Pain Physician. 2016;19:267‐280. 27228514

[62] Dworkin RH , Turk DC , Farrar JT , et al. Core outcome measures for chronic pain clinical trials: IMMPACT recommendations. Pain. 2005;113(1–2):9‐19. 15621359 10.1016/j.pain.2004.09.012

[63] Dworkin RH , Turk DC , McDermott MP , et al. Interpreting the clinical importance of group differences in chronic pain clinical trials: IMMPACT recommendations. Pain. 2009;146(3):238‐244. 19836888 10.1016/j.pain.2009.08.019

[64] Salmon J . High‐frequency spinal cord stimulation at 10 kHz for widespread pain: a retrospective survey of outcomes from combined cervical and thoracic electrode placements. Postgrad Med. 2019;131(3):230‐238. 30807247 10.1080/00325481.2019.1587564

[65] Simopoulos T , Yong RJ , Gill JS . Treatment of chronic refractory neuropathic pelvic pain with high‐frequency 10‐kilohertz spinal cord stimulation. Pain Pract. 2018;18(6):805‐809. 29106051 10.1111/papr.12656

[66] Arcioni R , Palmisani S , Mercieri M , et al. Cervical 10 kHz spinal cord stimulation in the management of chronic, medically refractory migraine: A prospective, open‐label, exploratory study. Eur J Pain. 2016;20(1):70‐78. 25828556 10.1002/ejp.692

[67] Silberstein S , Tfelt‐Hansen P , Dodick DW , et al. Guidelines for controlled trials of prophylactic treatment of chronic migraine in adults. Cephalalgia. 2008;28(5):484‐495. 18294250 10.1111/j.1468-2982.2008.01555.x

[68] Lambru G , Trimboli M , Palmisani S , Smith T , Al‐Kaisy A . Safety and efficacy of cervical 10 kHz spinal cord stimulation in chronic refractory primary headaches: a retrospective case series. J Headache Pain. 2016;17(1):66. 27393015 10.1186/s10194-016-0657-2 PMC4938814

[69] Amirdelfan K , Vallejo R , Benyamin R , et al. High‐frequency spinal cord stimulation at 10 kHz for the treatment of combined neck and arm pain: Results from a prospective multicenter study. Neurosurgery. 2019 10.1093/neuros/nyz495 PMC7360873 31792530

[70] Amirdelfan K , Vallejo R , Benyamin R , et al. A multicenter, prospective, clinical trial of high frequency spinal cord stimulation (HF‐SCS) at 10 kHz in the treatment of chronic upper limb and neck pain. Poster presented at: 22nd Annual Meeting of the North American Neuromodulation Society (NANS); January 17‐20, 2019; Las Vegas, NV.

[71] Burgher A , Kosek P , Surrett S , et al.10 kHz SCS for the treatment of chronic pain of the upper extremities: A post‐market observational study. Poster presented at: 22nd Annual Meeting of the North American Neuromodulation Society (NANS); January 17–20, 2019; Las Vegas, NV.

[72] Galan V , Chang P , Scowcroft J , Li S , Staats P , Subbaroyan J . A prospective clinical trial to assess high frequency spinal cord stimulation (HF‐SCS) at 10 kHz in the treatment of chronic intractable pain from peripheral polyneuropathy (PPN). Poster presented at: 22nd Annual Meeting of the North American Neuromodulation Society (NANS); January 17‐20, 2019; Las Vegas, NV.

[73] Gupta M , Scowcroft J , Kloster D , et al.Spinal cord stimulation (HF‐SCS) at 10 kHz for the treatment of chronic focal neuropathic post‐surgical pain: A prospective multicenter study. Poster presented at: 22nd Annual Meeting of the North American Neuromodulation Society (NANS); January 17–20, 2019; Las Vegas, NV.

[74] Kapural L , Paicius R , Gupta M , et al.Treatment of chronic abdominal pain with 10 kHz spinal cord stimulation in patients with diverse pain etiologies. Poster presented at: 22nd Annual Meeting of the North American Neuromodulation Society (NANS); January 17–20, 2019; Las Vegas, NV.

[75] Tate J , Stauss T , Li S , Subbaroyan J . A prospective, multi‐site, clinical trial of the high‐frequency spinal cord stimulation at 10 kHz (HF‐SCS) system in the treatment of chronic pelvic pain. Poster presented at: 22nd Annual Meeting of the North American Neuromodulation Society (NANS); January 17‐20, 2019; Las Vegas, NV.

[76] Argoff C , Mekhail N , Nasr C , et al. A prospective, randomized, controlled trial of high frequency spinal cord stimulation for the treatment of neuropathic limb pain from painful diabetic neuropathy: The SENZA‐PDN protocol. Poster presented at: 22nd Annual Meeting of the North American Neuromodulation Society (NANS); January 17‐20, 2019; Las Vegas, NV.

[77] Patel N , Wu C , Pilitsis J , et al.Taking spinal cord stimulation beyond failed back surgery syndrome: Design of a multicenter RCT for non‐surgical refractory back pain (NSRBP). Poster presented at: 22nd Annual Meeting of the North American Neuromodulation Society (NANS); January 17–20, 2019; Las Vegas, NV.

[78] Wesley S , Al‐Kaisy AA , Pang D , et al.Multicentre, double‐blind, randomised sham‐controlled trial: 10kHz high‐frequency SCS for chronic neuropathic low back pain (MODULATE‐LBP). Poster presented at: 22nd Annual Meeting of the North American Neuromodulation Society (NANS); January 17–20, 2019; Las Vegas, NV.

